# Reliability and Radiographic Correlation of the Foot Posture Index-6: A Multi-Rater Analysis in Symptomatic and Asymptomatic Individuals

**DOI:** 10.3390/diagnostics15101214

**Published:** 2025-05-12

**Authors:** Min Gyu Kyung, Yun Jae Cho, Jae Hee Lee, Min Seok Shin, Jay Hoon Park, Dong Yeon Lee

**Affiliations:** 1 Department of Orthopedic Surgery, Kyung Hee University Hospital at Gangdong, Seoul 05278, Republic of Korea; mgkyung@naver.com; 2 Department of Orthopedic Surgery, Han-il General Hospital, Seoul 01450, Republic of Korea; ckoko0@naver.com; 3 Department of Orthopedic Surgery, Dongdaemun Cham Teun Teun Hospital, Seoul 02530, Republic of Korea; ljaeh84@hanmail.net; 4 Department of Orthopedic Surgery, Seoul National University College of Medicine, Seoul 03080, Republic of Korea; curious0720@snu.ac.kr; 5 Department of Orthopedic Surgery, Seoul National University Hospital, Seoul 03080, Republic of Korea; jhpark1872@gmail.com

**Keywords:** foot, foot posture index, reliability, radiographic parameters, correlation

## Abstract

**Background/Objectives**: The foot posture index (FPI-6) is a practical clinical tool for evaluating standing foot posture using six specific criteria. Although widely used, its reliability and correlation with radiographic parameters remain uncertain. This study aimed to assess the inter-rater reliability of the FPI-6, in both asymptomatic individuals and patients with foot and ankle symptoms, and to examine its correlation with radiographic measurements. **Methods**: We included 40 asymptomatic male volunteers (group A) and 60 symptomatic patients (group B). Four raters independently assessed the FPI-6 scores, and inter-rater reliability was evaluated using the intraclass correlation coefficient. Radiographic parameters included the talocalcaneal angle (TCA) on anteroposterior (AP) and lateral views, talonavicular coverage angle (TNCA), AP talo-first metatarsal angle (TMA), hindfoot alignment angle (HAA), calcaneal pitch angle (CPA), and Meary’s angle (MA). Correlations between the FPI-6 and radiographic measurements were analyzed using Pearson’s correlation (*r*). **Results**: The FPI-6 showed good to excellent inter-rater reliability in both groups, with higher consistency in group B and among experienced raters. The total FPI-6 score significantly correlated with TNCA (*r* = 0.665), AP TMA (*r* = 0.453), lateral TCA (*r* = 0.369), MA (*r* = 0.570), and HAA (*r* = −0.773) (all *p* < 0.001). Group B demonstrated overall stronger correlations between the FPI-6 and radiographic measurements compared to group A (TNCA: 0.664 vs. 0.258; AP TMA: 0.542 vs. 0.139; lateral TCA: 0.492 vs. −0.101; MA: 0.544 vs. 0.172; and HAA: −0.712 vs. −0.374). **Conclusions**: With careful application, the FPI-6 is a reliable and valid tool for clinical assessment of foot posture, especially in settings without immediate access to radiographs.

## 1. Introduction

**Figure 1:**
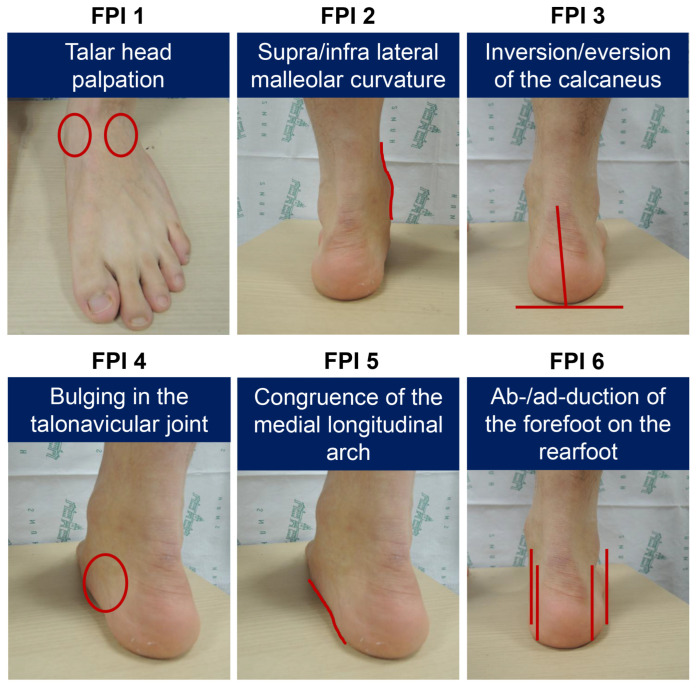
Each criterion of the foot posture index (FPI-6).

The foot posture index (FPI) was developed in response to the need for a quick, simple, and reliable method for diagnosing static foot posture in various clinical settings while minimizing the subjectivity of clinical evaluation methods [1,2]. Initially, the FPI was developed as an eight-item assessment, referred to as the FPI-8, and was later refined into a modified version known as the FPI-6 [3]. Based on three-dimensional observations, the FPI-6 includes six assessment criteria, each scored on a 5-point scale from −2 to +2, where negative values represent a more supinated foot posture and positive values indicate a more pronated foot posture [4,5]. Each criterion is as follows: FPI 1—talar head palpation, FPI 2—supralateral and infralateral malleolar curvature; FPI 3—inversion/eversion of the calcaneus; FPI 4—bulging in the talonavicular joint; FPI 5—congruence of the medial longitudinal arch; and FPI 6—abduction/adduction of the forefoot on the rearfoot (Figure 1). In previous studies, the FPI-6 has been utilized to evaluate foot posture as a risk factor for sports injuries and to investigate its relationship with plantar pressure, foot mobility, and foot kinematics [4,6,7,8,9].

The FPI-6 provides information on standing foot posture across various foot segments, through palpation and observation, allowing clinicians to perform evaluations without relying on complex measurement tools. However, its accuracy can be affected by the presence of soft tissue covering the foot’s skeletal structure, which is why radiographs remain the gold standard for assessing skeletal alignment. Therefore, it would be ideal if clinical measurements could accurately predict the angular measurements derived from radiographs. However, previous reports have demonstrated variable and conflicting results regarding the inter-rater reliability of the FPI [10,11,12] because it is influenced by various factors, such as different values depending on the rater’s experience, the age of the participant, and the presence of foot symptoms. Moreover, while previous studies have focused on the total FPI-6 scores, individual FPI-6 scores may differ [13,14]. Recently, studies correlating FPI-6 with radiographic measurements have also been conducted. Previous studies have primarily focused on elderly asymptomatic subjects, with an average age of 78.6 years, or have been conducted in specific populations such as children with flatfoot or adults with low arches [13,15,16]. According to Menz and Munteanu, in elderly asymptomatic subjects, the FPI demonstrated weaker correlations with radiographic parameters [13]. In contrast, in children with flatfoot, representative radiographic parameters such as the lateral talo-first metatarsal angle and calcaneal pitch were significantly correlated with the FPI-6 [15], whereas in adults with low arches, the arch angle was reported to correlate well with the FPI-6 [16]. However, there is a lack of studies that include both asymptomatic individuals and patients with various foot and ankle pathologies as study participants. Although the FPI-6 was originally developed to assess static foot posture and structure, we assumed that foot and ankle pathologies could secondarily alter foot posture in clinically meaningful ways. Pathological conditions such as flatfoot deformity, posterior tibial tendon dysfunction, hallux valgus (HV), and ankle osteoarthritis (OA) often lead to structural changes and compensatory postural adaptations of the foot. These changes are likely to be reflected in FPI-6 scores. Therefore, we believed that including both asymptomatic individuals and symptomatic patients would allow a broader evaluation of the FPI-6’s performance, not only in healthy foot structures but also in feet affected by various pathologies.

In addition, we believed that the level of clinical experience in assessing such patients using the FPI-6 is also an important factor. In previous studies, assessments were predominantly conducted by a single clinician or podiatrist [13,15], or involved only two physical therapists or athletic trainers, thereby limiting the range of rater experience. Therefore, we considered that incorporating raters with diverse levels of expertise could help identify differences according to experience.

Therefore, in this study, we aimed to evaluate the feasibility of using the FPI-6 for clinically assessing standing foot posture by (1) examining its reliability in both asymptomatic individuals and patients with foot and ankle conditions and (2) analyzing the correlation between individual FPI-6 criteria, as well as the total FPI-6 score, with radiographic measurements. We hypothesized that (1) the interrater agreement of the FPI-6 would be higher among experienced raters, (2) the individual components of the FPI-6 could be applied to assess specific segments of the foot in clinical settings for patients with foot and ankle symptoms, and (3) radiographic parameters that correspond to an individual FPI-6 would exist, such as the calcaneal pitch angle and congruence of the medial longitudinal arch.

## 2. Materials and Methods

### 2.1. Study Design and Participants

This prospective study received ethical approval from the Seoul National University Hospital Institutional Review Board (approval number: H-1010-047-335; approval date: 27 October 2010). Informed consent was obtained from all participants, and the study was conducted in compliance with the principles outlined in the Declaration of Helsinki.

Study participants were divided into two groups: asymptomatic individuals (group A) and patients with foot and ankle symptoms (group B). Each group served a distinct purpose: the asymptomatic group for establishing FPI-6 measurement reliability under controlled conditions, and the symptomatic group for investigating the FPI-6’s clinical correlation with radiographic measures.

Group A consisted of locally recruited asymptomatic males, aged between 20 and 28 years. By using a relatively homogeneous group (in terms of age and sex), we sought to reduce extraneous influences on the FPI-6 measurements. This helped ensure that any variability in FPI-6 scores was more likely due to the measurement process itself rather than demographic differences. In other words, a uniform asymptomatic group provided a stable baseline to evaluate how consistently the FPI-6 could be applied. The exclusion criteria were as follows: (1) experiencing subjective pain during walking, (2) a history of fractures or surgeries in the lower extremities, (3) any past neuromuscular disease affecting the foot or ankle, and (4) the presence of other disorders that cause difficulty in standing comfortably, such as tendinopathies or plantar fasciitis. After applying these criteria, 40 asymptomatic individuals were selected for group A.

For group B, we reviewed the medical records of patients who visited our outpatient clinic between August 2018 and May 2019 seeking treatment for foot and ankle symptoms accompanied by deformities. The exclusion criteria for group B were: (1) a history of prior surgery on the affected lower extremities and (2) an inability to maintain standing posture independently due to lower limb impairments, excluding those related to foot and ankle symptoms. Ultimately, 60 patients were included in group B for analysis. These patients included 5 patients with cavus foot [17], 29 with varus ankle OA [18], 9 with flat foot [19], four with valgus ankle OA [18], 4 with rheumatoid foot arthritis (RA) [20], and 9 with HV deformity [21].

### 2.2. FPI-6 Measurements

The FPI-6 was measured independently by four raters on the same day to reduce rater bias. Rater 1 was a physical therapist with over 10 years of experience in a general orthopedic clinic and 4 months of experience with FPI-6 measurements. Rater 2 was a physical therapist with 5 years of experience in general orthopedics and 4 months of FPI-6 measurement experience. Raters 1 and 2 were physical therapists who worked at the human motion analysis laboratory in our institute, where they were responsible for attaching skin markers for daily gait analyses. They were familiar with the musculoskeletal system, the surface anatomy of the foot, and palpation techniques. Raters 3 and 4 were relatively inexperienced, consisting of a medical school student and a junior orthopedic resident, respectively, with limited experience in measurements. Each participant was instructed to take a few steps forward, followed by marching in place for six to eight steps, before assuming a relaxed standing posture. This stance involved double-limb support with body weight evenly distributed between both feet, arms resting along the sides of the body, and the face facing forward. This position was maintained for approximately 1 min. Subsequently, six individual criteria were measured using a scale of −2, −1, 0, +1, or +2. The measurements included: FPI 1 for talar head palpation, FPI 2 for supralateral and infralateral malleolar curvature, FPI 3 for inversion/eversion of the calcaneus, FPI 4 for bulging in the talonavicular joint, FPI 5 for congruence of the medial longitudinal arch, and FPI 6 for abduction/adduction of the forefoot on the rearfoot. FPI 2, FPI 3, and FPI 6 were assessed from the posterior view, while FPI 4 and FPI 5 were assessed from the posterior oblique view. The detailed descriptions of each score from −2 to 2 for each FPI criterion are provided in a previous study [15].

### 2.3. Radiographic Measurements

All simple radiographs were obtained by the same radiologist using a consistent technique and the same digital radiography system (DigitalDiagnost; Philips Medical Systems, Andover, MA, USA). A standing foot anteroposterior (AP) view, lateral view, and hindfoot alignment view were each taken and used for analysis. The X-ray beam settings were 10 mAs, 60 kV, and a focus distance of 1 m for the AP and lateral views, and 20 mAs, 70 kV, with a focus distance of 1 m for the hindfoot alignment view. For the AP view of the foot, the X-ray beam was angled 10 degrees toward heel. For the lateral view, the X-ray beam was parallel to the floor, while the detector was positioned perpendicular to the floor. Participants were instructed to stand in an upright position with their second toe facing forward. For the hindfoot alignment view, the X-ray beam was angled 20 degrees caudally toward the ankle joint from the posterior aspect, with the detector positioned perpendicular at the anterior aspect of the foot. Participants were instructed to maintain an upright posture with weight equally distributed on both feet.

An orthopedic surgeon with over seven years of experience independently conducted the radiographic assessments, measuring the images in a blinded fashion. All measurements were performed using a picture archiving and communication system (PACS) (INFINITT PACS 7.0, Infinitt Healthcare, Seoul, Republic of Korea). Asymptomatic individuals were assessed on the right side, whereas patients were evaluated on the side with symptoms.

**Figure 2:**
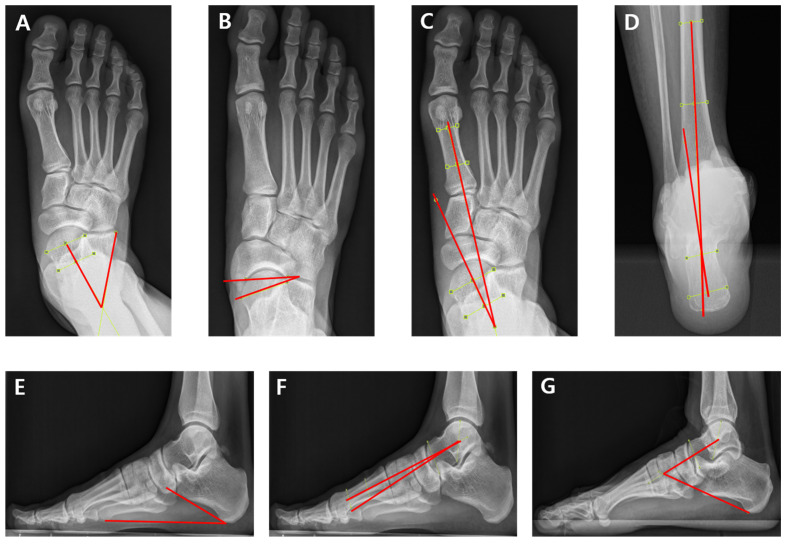
Radiographic measurements of the foot and ankle. (**A**) Anteroposterior talocalcaneal angle: the angle formed between a line bisecting the anterior surface of the talus and a line along the lateral border of the calcaneus, (**B**) talonavicular coverage angle: the angle between a line connecting the medial and lateral margins of the anterior talar articular surface and a line connecting the medial and lateral margins of the proximal navicular articular surface, (**C**) anteroposterior talo-first metatarsal angle: the angle between a line bisecting the anterior surface of the talus and the longitudinal axis of the first metatarsal, (**D**) hindfoot alignment angle: the angle formed by the long axis of tibia and the long axis of the calcaneus, (**E**) calcaneal pitch angle: the angle between a line drawn along the edge of the plantar soft tissue shadow and a line along the inferior border of the calcaneus, (**F**) Meary’s angle: the angle between the longitudinal axis of the talar head and the first metatarsal bone, and (**G**) lateral talocalcaneal angle: the angle formed by the long axis of the talus and a line along the inferior border of the calcaneus.

Seven radiographic parameters were measured: talocalcaneal angle (TCA) in both the AP and lateral views, talonavicular coverage angle (TNCA), AP talo-first metatarsal angle (TMA), hindfoot alignment angle (HAA), calcaneal pitch angle (CPA), and Meary’s angle (MA), as shown in Figure 2. These indices have demonstrated excellent reliability and strong discriminant validity [22,23,24]. Each radiographic parameter reflected the alignment of various foot segments. Specifically, the TCA, HAA, and CPA were chosen to evaluate hindfoot alignment, while the TNCA and MA were selected to assess midfoot alignment. The AP TMA was utilized to measure forefoot alignment.

### 2.4. Statistical Analysis

To assess the inter-rater reliability of the FPI-6 in both asymptomatic individuals and symptomatic patients, the intraclass correlation coefficient (ICC) of the type 2,1 was utilized. We interpreted an ICC value less than 0.5 as indicating poor reliability, an ICC between 0.5 and 0.75 as indicating good reliability, and an ICC of 0.75 or higher as indicating excellent reliability [25]. Pearson’s correlation analysis was used to assess the relationship between the FPI-6 and radiographic measurements, with the average FPI-6 scores from the two most experienced examiners selected for evaluation. The correlations were performed using both the total FPI-6 scores and the individual FPI-6 components. We assumed that using the data from the experienced raters would provide the most stable and accurate FPI-6 measurements, thereby yielding a more valid assessment of how FPI-6 correlates with radiographic alignment. We interpreted a correlation coefficient (*r*) of 0.5 or higher as having a low correlation, 0.75 or higher as having a moderate correlation, and 0.9 or higher as having a high correlation. Furthermore, descriptive statistics were used to analyze each pathology in group B, focusing on the distribution of both individual FPI-6 component scores and total FPI-6 scores. Statistical analyses were conducted using IBM SPSS Statistics version 27 (IBM Corp., Armonk, NY, USA), with a significance threshold set at *p* < 0.05.

## 3. Results

### 3.1. Demographic Data

The average age of the study participants was 23.8 years in group A and 61.9 years in group B (Table 1). Group A consisted of 40 males, whereas group B included 24 males and 36 females.

### 3.2. Reliability of the FPI-6

**Figure 3:**
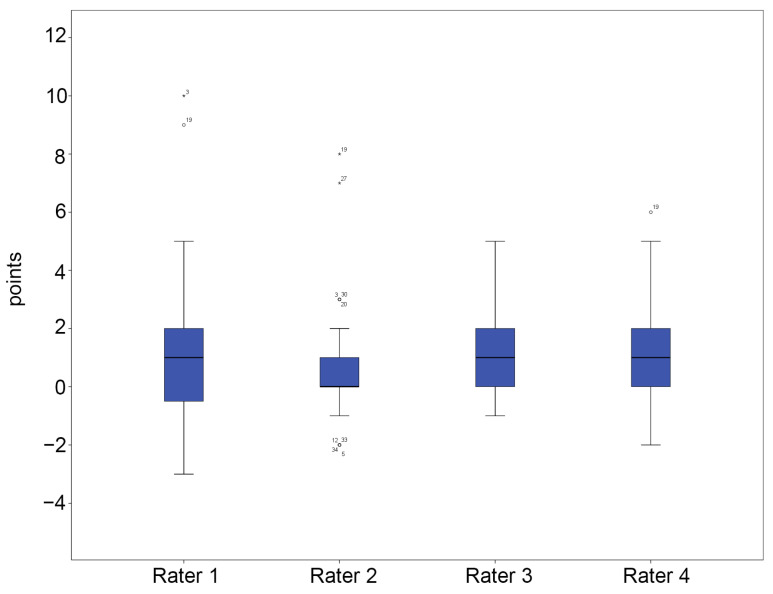
Five-number summary results of the FPI-6 score in group A. The vertical axis represents points. The central box represents the interquartile range (IQR), with the lower and upper edges corresponding to the first (Q1) and third quartiles (Q3), respectively. The line inside the box indicates the median value. Whiskers extend to the minimum and maximum values within 1.5 × IQR from the quartiles. Outliers beyond this range, if any, are plotted individually as dots or asterisks. Dots represent mild outliers (values more than 1.5 times the IQR) and asterisks represent extreme outliers (values more than 3 times the IQR).

**Figure 4:**
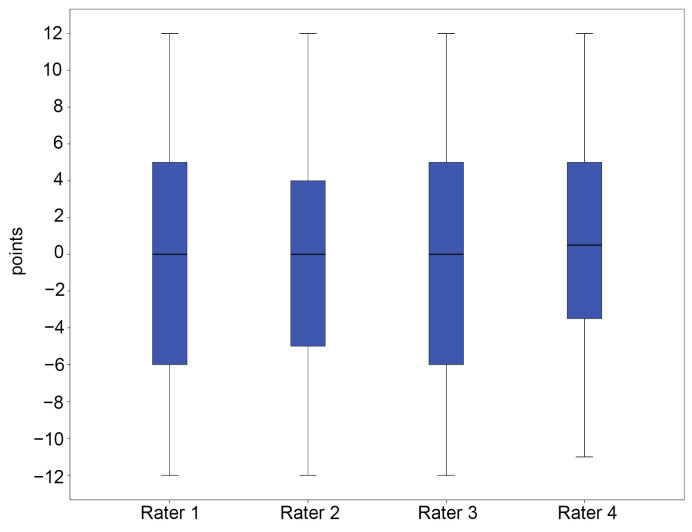
Five-number summary results of the FPI-6 score in group B. The vertical axis represents points. The central box represents the interquartile range (IQR), with the lower and upper edges corresponding to the first (Q1) and third quartiles (Q3), respectively. The line inside the box indicates the median value. Whiskers extend to the minimum and maximum values within 1.5 × IQR from the quartiles.

In group A, the mean total FPI-6 scores for each rater ranged from 0.73 to 1.15, indicating a slightly pronated foot posture (Figure 3 and Table S1). In contrast, group B showed a wider range of mean total FPI-6 scores, from −0.37 to 0.40, reflecting greater variability in foot posture, as anticipated (Figure 4 and Table S1). Remarkably, more outliers were observed in group A than in group B.

Regarding inter-rater reliability, the ICC of all raters in group A was 0.608 (95% confidence interval (CI): 0.454–0.851), indicating good repeatability, whereas that of all raters in group B was 0.878 (95% CI: 0.821–0.921), showing excellent repeatability (Table 2). The highest reliability was shown between raters 1 and 2, who were the most experienced, with scores of 0.787 (95% CI: 0.632–0.881) for group A and 0.938 (95% CI: 0.899–0.963) for group B. The two most experienced raters (rater 1 and rater 2) demonstrated an ICC of 0.927 (95% CI: 0.894–0.951) for a total of 100 participants, indicating excellent reliability.

### 3.3. Results of the Radiographic Measurements

**Figure 5:**
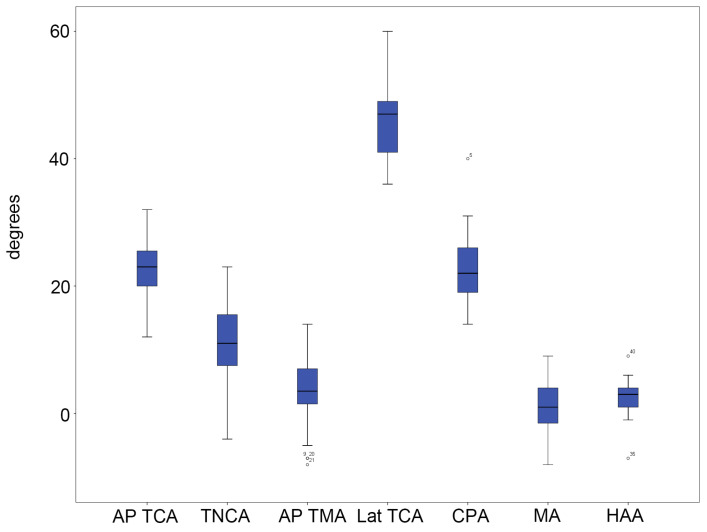
Five-number summary results of the radiographic measurements in group A. The vertical axis represents angles in degrees. The central box represents the interquartile range (IQR), with the lower and upper edges corresponding to the first (Q1) and third quartiles (Q3), respectively. The line inside the box indicates the median value. Whiskers extend to the minimum and maximum values within 1.5 × IQR from the quartiles. Outliers beyond this range, if any, are plotted individually as dots. Dots represent mild outliers (values more than 1.5 times the IQR) and asterisks represent extreme outliers (values more than 3 times the IQR). Abbreviations: AP, anteroposterior; FPI, foot posture index; TCA, talocalcaneal angle; TNCA, talonavicular coverage angle; TMA, talo-first metatarsal angle; CPA, calcaneal pitch angle; MA, Meary’s angle; HAA, hindfoot alignment angle; and Lat, lateral.

**Figure 6:**
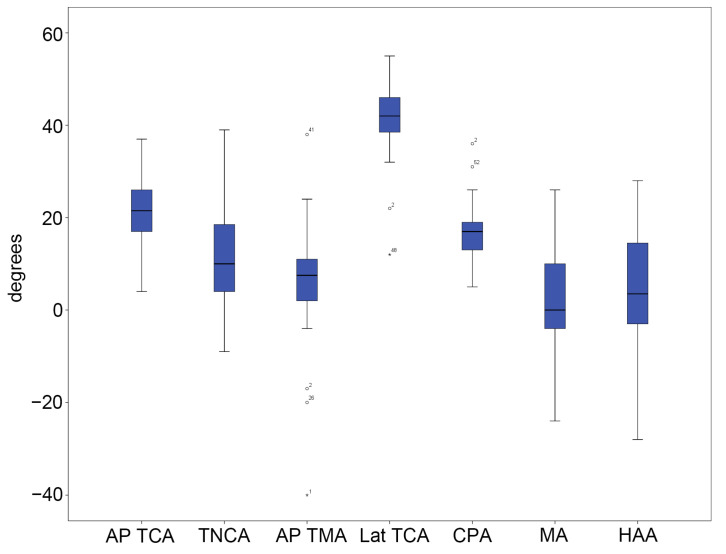
Five-number summary results of the radiographic measurements in group B. The vertical axis represents angles in degrees. The central box represents the interquartile range (IQR), with the lower and upper edges corresponding to the first (Q1) and third quartiles (Q3), respectively. The line inside the box indicates the median value. Whiskers extend to the minimum and maximum values within 1.5 × IQR from the quartiles. Outliers beyond this range, if any, are plotted individually as dots or asterisks. Dots represent mild outliers (values more than 1.5 times the IQR) and asterisks represent extreme outliers (values more than 3 times the IQR). Abbreviations: AP, anteroposterior; TCA, talocalcaneal angle; TNCA, talonavicular coverage angle; TMA, talo-first metatarsal angle; CPA, calcaneal pitch angle; MA, Meary’s angle; HAA, hindfoot alignment angle; and Lat, lateral.

In group A, the average and standard deviation for each radiographic parameter were as follows: AP TCA: 23.00° ± 4.15°; TNCA: 10.85° ± 6.20°; AP TMA: 3.38° ± 5.21°; lateral TCA: 46.03° ± 5.06°; CPA: 22.73° ± 4.88°; MA: 1.03° ± 4.02°; and HAA: 2.45° ± 2.56° (Figure 5 and Table S2). In group B, the average and standard deviation for each radiographic parameter were as follows: AP TCA: 21.47° ± 7.76°; TNCA: 10.39° ± 10.93°; AP TMA: 5.08° ± 13.04°; lateral TCA: 41.22° ± 7.92°; CPA: 17.03° ± 5.55°; MA: 2.94° ± 11.47°; and HAA: 4.19° ± 14.43° (Figure 6 and Table S2).

### 3.4. Correlation Between FPI-6 Scores and Radiographic Measurements

The total FPI-6 score significantly correlated with the TNCA (*p* < 0.001), AP TMA (*p* < 0.001), lateral TCA (*p* < 0.001), MA (*p* < 0.001), and HAA (*p* < 0.001). Notably, TNCA and HAA showed the strongest correlations, with coefficients of 0.665 (*p* < 0.001) and −0.773 (*p* < 0.001), respectively (Table 3). When comparing individual FPI-6 components with radiographic measurements, FPI 4 (bulging in the talonavicular joint) showed the strongest correlation with HAA (−0.748, *p* < 0.001), while FPI 5 (congruence of the medial longitudinal arch) was significantly correlated with TNCA (0.686, *p* < 0.001).

Group B demonstrated overall higher correlation coefficients (*r*) between FPI-6 and radiographic measurements compared to group A (TNCA: 0.664 vs. 0.258; AP TMA: 0.542 vs. 0.139; lateral TCA: 0.492 vs. −0.101; MA: 0.544 vs. 0.172; and HAA: −0.712 vs. −0.374) (Table 4 and Table 5).

### 3.5. Distribution of the FPI-6 Scores According to Foot Pathology in Group B

**Figure 7:**
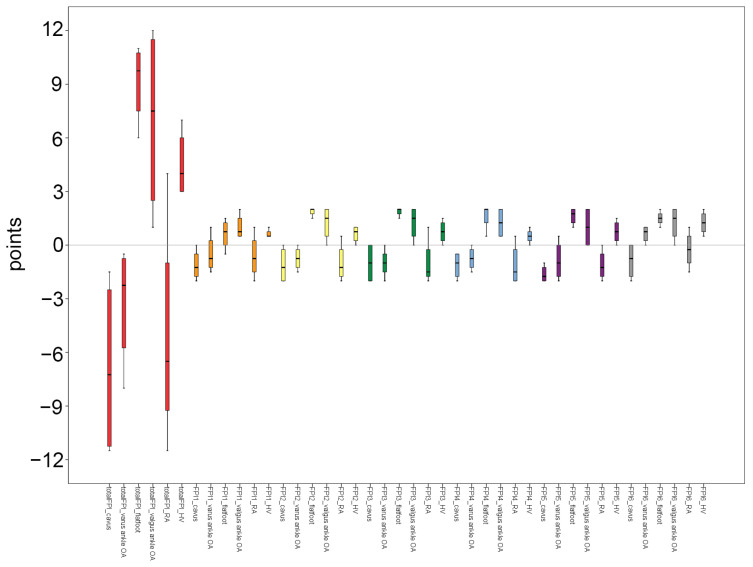
Five-number summary of FPI-6 scores according to foot pathology in group B. The vertical axis represents points. The central box represents the interquartile range (IQR), with the lower and upper edges corresponding to the first (Q1) and third quartiles (Q3), respectively. The line inside the box indicates the median value. Whiskers extend to the minimum and maximum values within 1.5 × IQR from the quartiles. Abbreviations: FPI, foot posture index; OA, osteoarthritis; RA, rheumatoid foot arthritis; and HV, hallux valgus (HV).

Not only did the individual FPI-6 scores yield negative values in the cavus foot, varus ankle OA, and RA patients, but the total FPI-6 score also yielded negative values under these conditions, whereas positive values were observed in patients with flat foot, valgus ankle OA, and HV deformity (Figure 7).

## 4. Discussion

In this study, we investigated the inter-rater reliability of the FPI-6 score and demonstrated good-to-excellent reliability in both asymptomatic individuals and patients with foot and ankle symptoms. Additionally, when compared to traditional radiographic measurements used to assess foot posture and alignment, it was found that there were variables that had a significant correlation with the FPI-6 score. Furthermore, when dividing foot pathologies into various subgroups and evaluating them using the FPI-6, the scores effectively reflected the characteristic postures of each disorder.

The mean total FPI-6 score in asymptomatic individuals ranged between 0.73 and 1.15, indicating a slightly pronated foot posture. This finding is consistent with those of previous studies that have demonstrated a tendency for normal feet to be pronated rather than completely neutral [26,27]. In contrast, the mean total FPI-6 score range for each rater in patients with foot and ankle symptoms was between −0.37 and 0.40. As expected, the patient group exhibited a wider range of FPI-6 scores due to the presence of various deformities associated with different pathologies. However, a few outliers were also identified in the asymptomatic group, indicating that the absence of symptoms does not necessarily correspond to normal foot and ankle postures.

Interestingly, the inter-rater reliability between raters 1 and 2, the most experienced evaluators, was excellent. Their familiarity with the musculoskeletal system, surface anatomy of the foot, and palpation techniques likely contributed to their ability to apply the FPI-6 criteria with high accuracy. However, a previous study indicated that even novice examiners with a background in musculoskeletal assessment could achieve reliable inter-rater results using the FPI-6 after minimal training [28]. In our study, the inter-rater reliability was relatively low among inexperienced examiners, such as medical school students and junior orthopedic residents, implying that extensive experience and training would be valuable for applying the FPI-6 in clinical situations, which is consistent with other studies [11,14].

Setting aside the proficiency of raters in FPI-6 measurement, the factors that can influence inter-rater discrepancy include the following: First, the FPI-6 manual lacks example illustrations for assigning intermediate scores. Therefore, if such ambiguous scoring ranges accumulate, the likelihood of significant score differences between raters will increase. Second, the illustration for FPI 4 in the manual depicts a foot area that does not precisely correspond to the talonavicular joint region, which could reduce inter-rater reliability, particularly in individuals without deformities [29]. Third, when assessors measure FPI-6 scores, they rely on observation and palpation. However, rather than directly palpating the bony structure, measurements can be significantly influenced by the soft tissue that covers it. Calluses, bunions, and edema are more prevalent in older individuals [30], which may lead to difficulties in visualizing the bony structures.

Importantly, our findings show that the FPI-6 demonstrated good-to-excellent inter-rater reliability in both group A and B. This means that even in the asymptomatic group with more subtle or “normal” foot posture variations, different raters largely agreed on the FPI-6 scores. Thus, the FPI-6 is not only reliable for classifying large, obvious deformities, but it also performs well in assessing more subtle differences in foot posture. This broad reliability underscores the utility of the FPI-6 across a spectrum of cases, from normal-aligned feet to markedly deformed feet. However, as the FPI-6 is based on visual observation and palpation of foot anatomy, when a patient has a clear deviation in foot posture (e.g., a significant flatfoot or high arch deformity), those features are more readily apparent to all examiners. This likely contributed to the slightly higher inter-rater agreement we observed in group B. In other words, obvious deformities provide more conspicuous cues, making it straightforward for multiple clinicians to agree on the FPI-6 score. Nevertheless, we believe this discrepancy does not diminish the clinical value of the FPI-6. The difference in reliability between the groups was not large and both were within a high reliability range. Therefore, we could say the FPI-6 is a valuable, practical tool for preliminary assessment in clinical settings.

We hypothesized that specific radiographic parameters would correspond to individual FPI-6 scores, such as a link between the congruence of the medial longitudinal arch and the CPA. However, bulging in the talonavicular joint showed the strongest correlation with the HAA, while the congruence of the medial longitudinal arch was more closely associated with TNCA. Although certain radiographic parameters showed some correlation with specific individual FPI-6 metrics, as hypothesized, the anticipated matches were not the strongest correlations observed. These findings were based on a combined analysis of all 100 participants, including both healthy and patient groups. When examined separately by dividing the participants into healthy and patient groups, the distinctions became more pronounced. The patient group demonstrated a stronger correlation between the FPI-6 scores and radiographic measurements. This could be attributed to the fact that asymptomatic participants may not exhibit distinct characteristics in radiographic parameters in contrast to those observed in the patient group. In the patient group, parameters such as the TNCA, AP TMA, lateral TCA, MA, and HAA demonstrated significant correlations with individual FPI-6 scores. Therefore, based on these correlation results, we believe that the individual FPI-6 could be utilized for specific segments of foot assessments in clinical settings for patients with foot and ankle symptoms and deformities. In particular, the distribution of FPI-6 scores according to foot pathology in the patient group also supports this, as both individual and total FPI-6 scores were negative (indicating supination) in conditions such as cavus foot, varus ankle OA, and RA, while the scores were positive (indicating pronation) in cases of flat foot, valgus ankle OA, and HV deformity. Therefore, there are areas of foot and ankle pathology where clinical assessment can be aided through FPI-6 scoring.

Furthermore, the FPI-6 can be a practical tool for assessing the posture of different foot and ankle segments during physical examination, especially in settings where radiographic imaging is not readily available or when evaluating new patients in an outpatient clinic before ordering radiographic studies. We believe that it allows clinicians to intuitively assess foot posture and anticipate corresponding radiographic parameters. Although our study showed some variability in FPI-6 scoring depending on clinical experience, with proper training, it can serve as a simple and useful tool for effective application in clinical practice.

This study has several distinguishing features compared to previous research. First, to assess the reliability of the FPI-6, we included four independent raters with varying levels of experience: two physical therapists with different years of clinical experience, as well as a medical student and a junior orthopedic resident with a relatively lower amount of experience. While previous studies have also examined the reliability of the FPI-6, they were predominantly conducted by a single clinician or podiatrist [13,15], or involved only two physical therapists, graduate-level students, or athletic trainers, respectively [10,16,28]. By incorporating raters with diverse levels of expertise, our study provides insight into how FPI-6 scores may vary when applied in real clinical settings. Second, we evaluated the correlation between FPI-6 scoring and radiographic parameters in both asymptomatic individuals and patients with various foot and ankle conditions commonly encountered in clinical practice. Previous studies have either focused solely on asymptomatic individuals [13] or examined the application of the FPI-6 in specific conditions, such as flatfoot [15,16]. We believe that including a broad range of clinically relevant conditions offers a valuable opportunity to assess the reliability of the FPI-6 across diverse foot and ankle deformities. Lastly, we included seven radiographic parameters that have been previously established as reliable and valid measures [22,23,24]. Each of these parameters reflects the alignment of various foot segments, allowing us to assess the relationship between individual FPI-6 criteria and radiographic findings. This, in turn, provided a comprehensive evaluation of static foot posture.

## 5. Limitations and Future Work

This study had several limitations. First, the distributions of foot and ankle pathologies in the patient group were uneven. Apart from the varus ankle OA patient subgroup, the other subgroups included only a small number of individuals. It is important to note that the proportion of patients presenting to our institution may vary depending on the specific condition. Nevertheless, caution should be exercised when extrapolating these results to clinical practice. Future studies with adequate sample sizes for each subgroup are warranted to enable more robust subgroup analyses. Second, the sex and age of the participants were not controlled. Group A consisted solely of young males, whereas group B consisted of comparatively older males and females. This age and sex discrepancy may limit the repeatability and correlation of the findings. However, we believe this issue is minor because our aim was not to compare the absolute FPI-6 scores between groups, and the age range for the foot and ankle pathologies mentioned in the text is not exclusively young. Third, we only assessed inter-rater reliability. Intra-rater reliability, which evaluates the consistency of repeated scoring by the same rater, is also an important aspect. Future studies should take this into consideration. Finally, while the repeatability of the FPI-6 was confirmed by each of the four raters, the radiographic parameters were analyzed by a single orthopedic surgeon. This could lead to potential bias or a decrease in reliability. Therefore, additional studies are needed to investigate the impact of these potential confounding factors and to address the limitations outlined above.

## 6. Conclusions

The FPI-6 is a reliable clinical tool for assessing foot posture in both asymptomatic individuals and patients with foot and ankle symptoms, provided it is performed by trained examiners. In addition, the correlations between total and individual FPI-6 scores and specific radiographic measurements may help clinicians anticipate corresponding radiographic parameters and alignments.

## Figures and Tables

**Table 1:** Participants’ demographic data.

	Group A (*n* = 40)	Group B (*n* = 60)
Age, years	23.8 ± 1.9 (21–28)	61.9 ± 15.3 (9–83)
Sex, numbers	Male 40	Male 24, Female 36
Height, cm	174.6 ± 4.7 (163.0–186.0)	158.3 ± 8.9 (132.0–177.7)
Weight, kg	72.6 ± 9.7 (48.6–100.9)	65.5 ± 12.1 (30.6–89.9)
BMI, kg/m^2^	23.8 ± 3.2 (16.3–32.9)	26.1 ± 4.2 (16.1–40.8)

Data are presented in mean ± standard deviation (range).

**Table 2:** Inter-rater reliability of the FPI-6 by the intra correlation coefficient.

	Group A (*n* = 40)	Group B (*n* = 60)
Rater 1 vs. Rater 2	0.787	0.938
Rater 1 vs. Rater 3	0.583	0.962
Rater 1 vs. Rater 4	0.471	0.809
Rater 2 vs. Rater 3	0.637	0.938
Rater 2 vs. Rater 4	0.535	0.772
Rater 3 vs. Rater 4	0.601	0.824
Total	0.608	0.878

**Table 3:** Correlation between the FPI-6 and radiographic measurements in all 100 participants.

	AP TCA	TNCA	AP TMA	Lat TCA	CPA	MA	HAA
FPI 1	0.136	0.620 *	0.492 *	0.399 *	−0.052	0.530 *	−0.713 *
FPI 2	0.109	0.602 *	0.398 *	0.317 *	−0.066	0.505 *	−0.738 *
FPI 3	0.127	0.583 **	0.339 **	0.356 **	0.002	0.467 *	−0.726 **
FPI 4	0.072	0.573 **	0.358 **	0.323 **	−0.052	0.485 **	−0.748 **
FPI 5	0.166	0.686 **	0.438 **	0.318 **	−0.263 **	0.658 **	−0.664 **
FPI 6	0.168	0.597 **	0.503 **	0.346 **	−0.095	0.512 **	−0.658 **
Total FPI-6	0.140	0.665 **	0.453 **	0.369 **	−0.096	0.570 **	−0.773 **

Note: The results are expressed as Pearson’s correlation coefficients (*r*). * denotes *p* < 0.05 and ** denotes *p* < 0.001. Abbreviations: AP, anteroposterior; FPI, foot posture index; TCA, talocalcaneal angle; TNCA, talonavicular coverage angle; TMA, talo-first metatarsal angle; CPA, calcaneal pitch angle; MA, Meary’s angle; HAA, hindfoot alignment angle; and Lat, lateral.

**Table 4:** Correlation between the FPI-6 and radiographic measurements in group A (*n* = 40).

	AP TCA	TNCA	AP TMA	Lat TCA	CPA	MA	HAA
FPI 1	−0.086	0.381 *	0.048	−0.255	−0.337 *	0.328 *	−0.267
FPI 2	0.031	0.159	−0.164	−0.124	−0.097	0.114	−0.134
FPI 3	−0.100	0.206	0.060	−0.326 *	−0.369 *	0.281	−0.177
FPI 4	−0.091	0.320 *	0.027	−0.091	−0.148	0.217	−0.171
FPI 5	−0.095	0.130	−0.176	−0.021	−0.144	0.228	−0.213
FPI 6	0.046	0.560 **	0.198	−0.337 *	−0.487 **	0.518 **	−0.268
Total FPI-6	−0.156	0.258	0.139	−0.101	−0.200	0.172	−0.374 *

Note: The results are expressed as Pearson’s correlation coefficients (*r*). * denotes *p* < 0.05 and ** denotes *p* < 0.001. Abbreviations: AP, anteroposterior; FPI, foot posture index; TCA, talocalcaneal angle; TNCA, talonavicular coverage angle; TMA, talo-first metatarsal angle; CPA, calcaneal pitch angle; MA, Meary’s angle; HAA, hindfoot alignment angle; and Lat, lateral.

**Table 5:** Correlation between the FPI-6 and radiographic measurements in group B (*n* = 60).

	AP TCA	TNCA	AP TMA	Lat TCA	CPA	MA	HAA
FPI 1	0.160	0.713 **	0.500 **	0.455 **	−0.138	0.605 **	−0.801 **
FPI 2	0.136	0.705 **	0.591 **	0.465 **	−0.168	0.601 **	−0.765 **
FPI 3	0.122	0.672 **	0.456 **	0.391 **	−0.127	0.556 **	−0.780 **
FPI 4	0.143	0.629 **	0.388 **	0.404 **	−0.065	0.514 **	−0.761 **
FPI 5	0.080	0.641 **	0.421 **	0.355 **	−0.133	0.526 **	−0.777 **
FPI 6	0.192	0.712 **	0.469 **	0.480 **	−0.247	0.678 **	−0.716 **
Total FPI-6	0.237	0.664 **	0.542 **	0.492 **	−0.033	0.544 **	−0.712 **

Note: The results are expressed as Pearson’s correlation coefficients (*r*). ** denotes *p* < 0.001. Abbreviations: AP, anteroposterior; FPI, foot posture index; TCA, talocalcaneal angle; TNCA, talonavicular coverage angle; TMA, talo-first metatarsal angle; CPA, calcaneal pitch angle; MA, Meary’s angle; HAA, hindfoot alignment angle; and Lat, lateral.

## Data Availability

The datasets generated during and/or analyzed during the current study are available from the corresponding author on reasonable request.

## Supplementary Materials

The following supporting information can be downloaded at: https://www.mdpi.com/article/10.3390/diagnostics15101214/s1, Table S1: Total FPI-6 score for each rater in groups A and B. Table S2: Radiographic measurements in groups A and B.

## Abbreviations

The following abbreviations are used in this manuscript:

FPI	foot posture index
OA	osteoarthritis
RA	rheumatoid foot arthritis
HV	hallux valgus
TCA	talocalcaneal angle
AP	anteroposterior
TNCA	talonavicular coverage angle
TMA	talo-first metatarsal angle
HAA	hindfoot alignment angle
CPA	calcaneal pitch angle
MA	Meary’s angle
ICC	intraclass correlation coefficient
CI	confidence interval
